# Recognizing facial expressions of emotion amid noise: A dynamic advantage

**DOI:** 10.1167/jov.24.1.7

**Published:** 2024-01-10

**Authors:** Anne-Raphaëlle Richoz, Lisa Stacchi, Pauline Schaller, Junpeng Lao, Michael Papinutto, Valentina Ticcinelli, Roberto Caldara

**Affiliations:** 1 Eye and Brain Mapping Laboratory (iBMLab), Department of Psychology, University of Fribourg, Fribourg, Switzerland

**Keywords:** facial expressions of emotion, static, dynamic, parametric noise, psychophysics

## Abstract

Humans communicate internal states through complex facial movements shaped by biological and evolutionary constraints. Although real-life social interactions are flooded with dynamic signals, current knowledge on facial expression recognition mainly arises from studies using static face images. This experimental bias might stem from previous studies consistently reporting that young adults minimally benefit from the richer dynamic over static information, whereas children, the elderly, and clinical populations very strongly do (Richoz, Jack, Garrod, Schyns, & Caldara, 2015, Richoz, Jack, Garrod, Schyns, & Caldara, 2018b). These observations point to a near-optimal facial expression decoding system in young adults, almost insensitive to the advantage of dynamic over static cues. Surprisingly, no study has yet tested the idea that such evidence might be rooted in a ceiling effect. To this aim, we asked 70 healthy young adults to perform static and dynamic facial expression recognition of the six basic expressions while parametrically and randomly varying the low-level normalized phase and contrast signal (0%–100%) of the faces. As predicted, when 100% face signals were presented, static and dynamic expressions were recognized with equal efficiency with the exception of those with the most informative dynamics (i.e., happiness and surprise). However, when less signal was available, dynamic expressions were all better recognized than their static counterpart (peaking at ∼20%). Our data show that facial movements increase our ability to efficiently identify emotional states of others under the suboptimal visual conditions that can occur in everyday life. Dynamic signals are more effective and sensitive than static ones for decoding all facial expressions of emotion for all human observers.

## Introduction

Human facial movements transmit a wealth of dynamic signals that readily provide crucial information about other people's emotional states. The temporal dynamics of facial expressions of emotion are finely optimized to hierarchically transmit biologically rooted and socially adaptive signals over time (Jack, Garrod, & Schyns, 2014). In everyday life, we are flowed by dynamic emotional signals steadily stimulating the visual system over the course of social interactions. As a consequence, the frequency of exposure to dynamic faces is markedly higher compared with static ones. Yet, most studies investigating facial expression recognition (FER) have relied on the sole use of static face images; thus, very little is known about how the human brain decodes and processes dynamic facial expressions of emotion. This experimental bias might stem from previous studies consistently reporting that young adults minimally benefit from the richer dynamic over static information, whereas children, the elderly and, clinical populations very strongly do (Richoz, Jack, Garrod, Schyns, & Caldara, 2015; Richoz, Jack, Garrod, Schyns, & Caldara, 2018b). Dynamic face stimuli are also more difficult to control; experiments using dynamic faces take longer, and there are fewer databases available for dynamic compared with static faces. In fact, as previously pointed out by Dobs, Bulthoff, & Schultz, (2018), controlling the role of motion and form information in dynamic ecological natural stimuli is challenging. Point lights can provide and test the importance of facial motion information, but they are highly unnatural. Morphing techniques might help to control motion while approximately preserving form. However, as noted by Dobs et al. (2018), they only represent a coarse linear approximation of natural face motion, which might lead to less accurate emotion recognition than their natural counterparts (Cosker, Krumhuber, & Hilton, 2010; Korolkova, 2018; Wallraven, Breidt, Cunningham, & Bülthoff, 2018). Synthetic faces might help to solve this challenge, but they suffer in terms of ecological validity. Despite these methodological challenges, the lack of studies using dynamic natural faces is still surprising because evolutionary and ontogenetic perspectives would posit an advantage in processing dynamic faces for all human observers.

From an evolutionary perspective, humans have had more extensive experience with dynamic faces as the perception of static faces emerged only recently in human history, with the first existing portraits originating in ancient Egypt approximately 5,000 years ago. For hundreds of centuries, this form of art was a privilege of the nobles and powerful. Although exposure to paintings and statues increased throughout history, it is only during the last century that humans have become more and more confronted with static face images with the advent of photography and the rapid expansion of digital technologies. Critically, daily exposures to static faces occurred only during the last 20 years with the use of smartphones and the diffusion of pictures and selfies throughout social networks and media content. However, during the very last few years, dynamic social media messages are massively overtaking static ones over the internet (i.e., Instagram, TikTok). The exposure to static face images in social media remains limited compared with dynamic face signals. Moreover, from an ontogenetic perspective, infants are mostly deprived of any exposure to static faces during their first year of life. Infants rapidly tune to cultural differences in facial expressions of emotion based on learning cues in dynamic signals (Geangu et al., 2016). Although daily exposure and evolutionary and ontogenetic perspectives would predict an advantage in processing dynamic over static facial expressions, this question remains unclear.

To date, a relatively modest number of studies have investigated this question, yielding contradictory findings (for a review, see Alves, 2013; Fiorentini & Viviani, 2011; Kätsyri, 2006; Krumhuber, Kappas, & Manstead, 2013). Although some studies did not find a dynamic advantage in healthy young adults (e.g., Fiorentini & Viviani, 2011; Gold et al., 2013; Kätsyri & Sams, 2008), an advantage was observed in clinical (e.g., Atkinson, Dittrich, Gemmell, & Young, 2014; Kan, Mimura, Kamijima, & Kawamura, 2004; Schaefer, Baumann, Rich, Luckenbaugh, & Zarate, 2010) and neuropsychological populations (Adolphs, Tranel, & Damasio, 2003; Humphreys, Donnelly, & Riddoch, 1993; Richoz et al., 2015). For example, Yitzhak, Gilaie-Dotan, & Aviezer, (2018) investigated emotion recognition with patient L.G., a single case of developmental visual agnosia and prosopagnosia. Their results revealed improved recognition scores when subtle nonstereotypical expressions were presented with dynamic compared with static faces. We observed similar findings in another neuropsychological study examining FER in patient P.S., a pure case of acquired prosopagnosia with bilateral occipitotemporal lesions anatomically sparing the regions critical for decoding facial expressions. Although patient P.S. was selectively impaired in categorizing several static expressions, her performance was within the average range with dynamic faces (Richoz et al., 2015). In contrast with L.G.’s relatively poor dynamic advantage, the gain for patient P.S. was prominent; she reached maximum accuracy for all dynamic expressions except for fear. Her impaired performance for static expressions can be accounted for by a suboptimal use of facial information with static faces, for which she only relies on the lower part of the face (Fiset et al., 2017). More important, P.S.’ peculiar brain lesions point to the existence of distinct cortical pathways for processing static and dynamic face information (Bernstein, Erez, Blank, & Yovel, 2018; Duchaine & Yovel, 2015; Fox, Iaria, & Barton, 2009; Pitcher, Dilks, Saxe, Triantafyllou, & Kanwisher, 2011). P.S.’s advantage for the recognition of dynamic facial expressions might rely on an intact functional cortical pathway directly connecting the early visual cortex to the posterior superior temporal sulcus, and subsequent processing in the anterior superior temporal sulcus (Richoz et al., 2015).

Pursuing these observations, we recently investigated FER across the lifespan from 5 to 96 years of age. We aimed to empirically probe the hypothesis that the dynamic advantage occurs only in populations with fragile face processing systems—with difficulties extracting facial information—such as very young children, whose system is yet to fully mature, and elderly populations whose system is declining (Richoz et al., 2018b). Our findings supported our hypothesis; we observed a clear dynamic advantage in both young children and the elderlies, whereas young adults exhibited only a minimal benefit for dynamic stimuli and only for a very limited number of expressions. These findings are consistent with previous behavioral studies revealing that the latter population does not benefit from the presentation of dynamic signals (Fiorentini & Viviani, 2011; Gold et al., 2013; Kätsyri & Sams, 2008). For example, Fiorentini and Viviani (2011) reported similar identification accuracies and reaction times for both static and dynamic expressions in a study using a threshold model with morphed expressions. Using an ideal observer approach to objectively assess how much information is carried by their stimuli across varying tasks and conditions, Gold et al. (2013) further evidenced identical recognition scores for static, dynamic, and even shuffled and reversed expressions in which movie frames were randomly presented or temporally reversed. Altogether, these findings suggest a near-optimal FER system in healthy young adults—as also argued by fast periodic visual stimulation studies that revealed specific brain responses to basic emotional facial expressions at a single glance (e.g., Dzhelyova, Jacques, & Rossion, 2017; Poncet, Baudouin, Dzhelyova, Rossion, & Leleu, 2019).

In optimal situations and with expressions of high intensity, the visual system of healthy young adults seems thus to be powerful enough to efficiently categorize static emotional expressions, leaving only little scope for improvement when dynamic faces are shown. As suggested in previous reviews (Dobs et al., 2018; O'Toole, Roark, & Abdi, 2002), dynamic cues are beneficial only in suboptimal visual conditions in which the facial information is limited. This has been shown in several studies in healthy young adults with degraded (Ambadar, Schooler, & Cohn, 2005; Bould & Morris, 2008; Bould, Morris, & Wink, 2008) or blurred stimuli (Ehrlich, Schiano, & Sheridan 2000; Kätsyri & Sams, 2008; Wallraven, Breidt, Cunningham, & Bülthoff, 2008).

For example, Wallraven et al. (2008) revealed that dynamic faces led to more accurate recognition scores when facial features, such as texture, shape, or motion information, were gradually degraded over four different blur levels. In a further study, Cunningham and Wallraven (2009) revealed an overall better recognition performance for dynamic over static expressions when the faces were presented with varying spatial information (point-light, wireframe, and full-surface faces). Although performance was near chance level for static faces in the point-light condition, the recognition of dynamic expressions was overall higher and remarkably similar across all three experimental manipulations. This report suggests that motion information can mitigate the negative consequences of degrading, blurring, or changing the texture of face and shape information. The facilitative effects driven by dynamic stimuli were further observed in studies using complex stimuli such as nonstereotypical (Yitzhak et al., 2018), conversational (Cunningham &Wallraven, 2009), subtle (Ambadar et al., 2005; Bould et al., 2008), or genuine versus deliberate expressions (Namba, Kabir, Miyatani, & Nakao, 2018; Zloteanu, Krumhuber, & Richardson, 2018). With these expressions, additional information provided by moving faces might be necessary to compensate for the ambiguity elicited by complexity or the lack of intensity (Krumhuber et al., 2013).

To sum up, previous studies either failed to report a dynamic advantage in healthy young adults, which is possibly reflecting a ceiling effect owing to a near-optimal facial expression decoding system in this population or reported the existence of very limited dynamic advantage for FER in healthy young adults, restricted to situations in which FER is disturbed through stimulus manipulation. This observation raises the question of how much signal is needed to elicit a dynamic advantage. To clarify this question, we parametrically and randomly manipulated the signal (i.e., phase coherence) of the facial expressions presented to the observers to determine how much signal they need to recognize the emotional expressions accurately (from 0% to 100%). We implemented a similar approach to the one we used in several previous studies (Bayet et al., 2017; Rodger, Vizioli, Ouyang, & Caldara, 2015; Rodger, Lao, & Caldara, 2018; Rodger et al., 2021; Wyssen et al., 2019) yet adapted it to make it more suitable for dynamic faces. More specifically, we parametrically manipulated the phase coherence (i.e., the quantity of signal) by using an adaptation of the weighted mean phase technique by Dakin, Hess, Ledgeway, & Achtman, (2002) (see also Rainer, Augath, Trinath, & Logothetis, 2001; Rousselet, Pernet, Bennett, & Sekuler, 2008). Importantly, we implemented an approach equating all the dynamic and static stimuli for their low-level image properties (i.e., spatial frequency spectrum and contrast). Each stimulus was then characterized by its level of signal with 0% corresponding to completely de-phased images and 100% to natural images (100% of phase coherence). On each trial, the level of signal was determined by a uniform or adaptive sampling, with the latter being based on participants’ previous performance. Seventy-one healthy young adults performed FER tasks of the six basic expressions (i.e., anger, disgust, fear, happiness, sadness, surprise) in static and dynamic conditions. We relied on a database of the six basic static and dynamic facial expressions of emotion) created by (Gold et al., 2013). Our methodological choice was driven by the fact that these authors used an ideal observer model to objectively measure the amount of *low-level* physical information in the stimuli, as previous studies did not control for this factor (e.g., Ambadar et al., 2005; Bould et al., 2008; Cunningham & Wallraven, 2009; Fiorentini & Viviani, 2011). Importantly, this database has been successfully used in several studies (Richoz et al., 2018a; Richoz et al., 2018b) and led to similar FER performance profiles as those observed with the seminal Pictures of Facial Affect database developed by Ekman and Friesen, 1976; Ekman and Friesen, 1978, corroborating its validity.

## Methods

### Participants

Seventy-one healthy young adults participated in the study (*M* = 20.9 years; *SD* = 1.7, 16 males). All participants had normal or corrected-to-normal visual acuity and did not suffer from neurological, developmental, or psychiatric disorders. They were all Caucasian and have always lived in a Caucasian culture. We decided to control for the ethnicity of our participants, given that cultural factors have an influence on FER (Jack, Caldara, & Schyns, 2012; Jack, Garrod, Yu, Caldara, & Schyns, 2012), and this can be observed already very early in infancy (Geangu et al., 2016). Most of our participants were university students (67) and received course credits for their participation. Before starting the experiment, all participants signed a consent form explaining the main goals of the study. The Ethical Committee of the Department of Psychology of the University of Fribourg in Switzerland approved the current study. All participants provided written informed consent in accordance with the Declaration of Helsinki.

### Stimuli

We used a set of static and dynamic stimuli created by Gold et al. (2013). They generated their database by asking eight actors (four females) to perform the six basic facial expressions of emotion (i.e., anger, disgust, fear, happiness, sadness, and surprise) (Ekman & Friesen, 1976). All dynamic expressions started with a neutral face and naturally evolved into a fully articulated expression over the course of 30 frames. The apex of each expression (i.e., the point at which an expression reached its highest state) was determined by two raters. Each expression was presented for 1000ms at a frame rate of 30 frames/second, and all expressions reached their apex within 30 frames. If an expression was fully articulated before reaching 30 frames or if head movements or other artifacts occurred, one to four supplementary apex frames were added to the dynamic movie to reach the duration of 1s (this happened only for seven out of 48 movies; for more details, see Gold et al., 2013). All dynamic expressions were presented as front-view grayscale faces cropped at the hairline to show only the internal facial features to the observers. In addition, the faces were displayed within an oval frame, located in the center of a dark grey uniform background. The edges of the oval frame were slightly blurred to create a gradual transition between the facial expressions and the grey-colored background (Gold et al., 2013). The faces subtended a visual angle of 12° at a viewing distance of 65 cm from the screen and the images were 768 pixels in height and 768 pixels in width.

**Figure 1.**
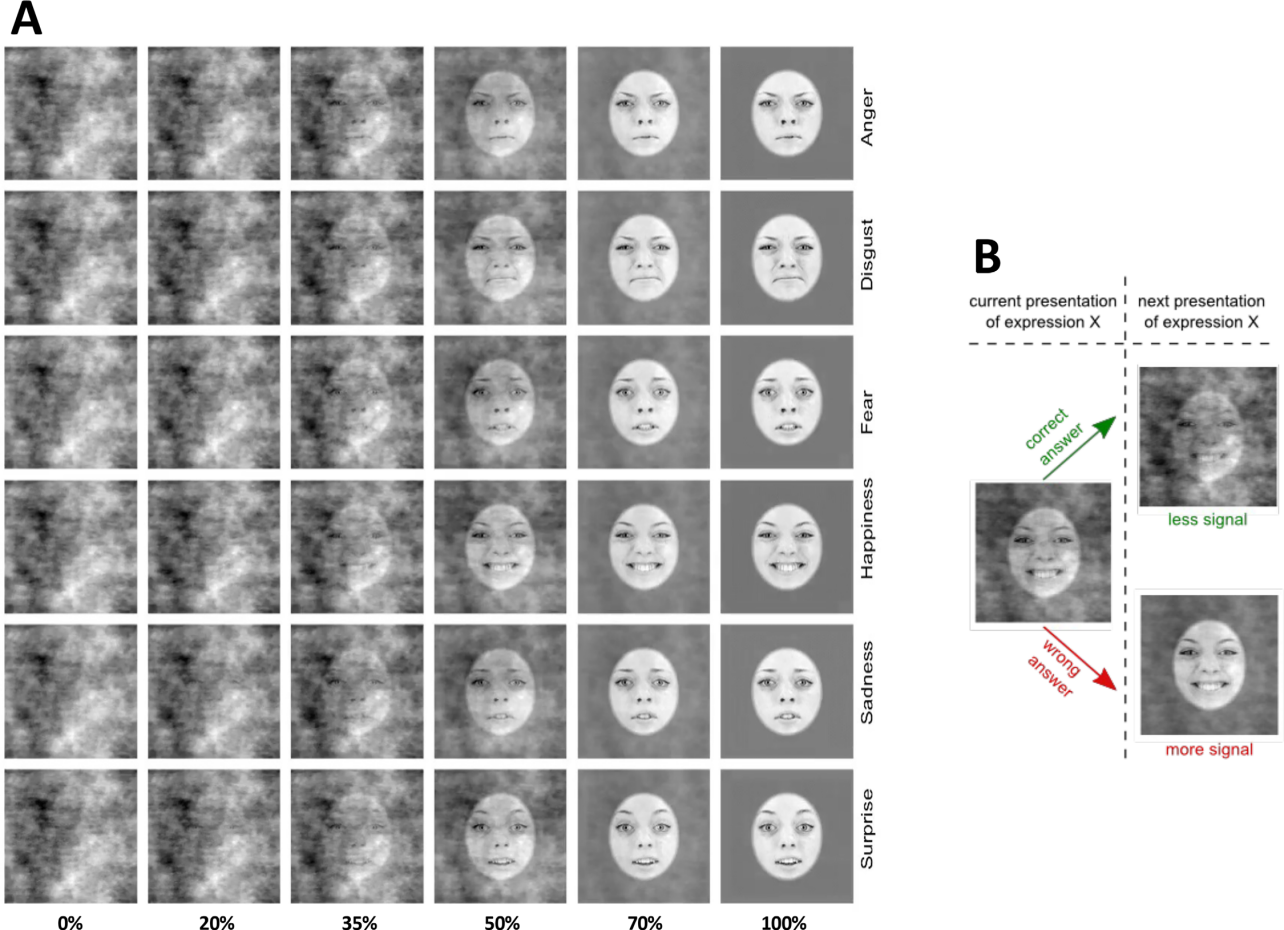
(**A**) Static examples of one identity expressing the six basic emotions at different levels of phase signal. The rows represent the six basic expressions (anger, disgust, fear, happiness, sadness, and surprise), and the columns the different levels of signal (0%, 20%, 40%, 50%, 60%, 80%, and 100%). We adapted the stimuli with permission from Gold et al. (2013). Further illustrative video examples can be seen with the following links for the static condition—Movie 1 and the dynamic condition Movie 3. (**B**) A schematic illustration of the adaptive sampling approach.

The static facial expression movies were created by replicating the final frame of each dynamic movie 30 times in a row (see Figure 1; video examples of the stimuli can be found under the links).

We used the SHINE toolbox (Willenbockel et al., 2010) to normalize all stimuli for their low-level properties (spatial frequency and contrast) and the amount of energy sampled over time, even for the static facial expressions (see Richoz et al., 2018b). This procedure allowed us to ensure that all the frames for all the faces in all conditions had equal low-level properties over time.

The stimuli were presented to the observers with varying levels of phase coherence (i.e., signal). The percentage of phase coherence varied between 100% for natural images to 0% for completely de-phased images. To parametrically manipulate the phase coherence, we adapted the weighted mean phase technique by Dakin et al., 2002; see also (Rainer et al., 2001; Rousselet et al., 2008) so that our faces were dephased while contrast and luminance were maintained constant across all levels of signal. More specifically, the final image (*I_final_*) shown to observers was obtained by combing an original image with 100% phase coherence (*I_initial_*) with a noise image (*I_noise_*) as it follows:

$$\begin{array}{c} I_{final}=\;w\;I_{initial}+\left(1-w\right)I_{noise}\;w\;\left[0,\;1\right] \end{array}$$

where *w* defines the degree of phase coherence of the final image.

*I_noise_* was built through the inverse fast Fourier transform using the same amplitude as the original image (*a_initial_*), but a different phase (φ_*noise*_). Specifically, these two components of the noise image were obtained using the following equations:

$$\begin{array}{ccc} \;\;\;\;\;\;\;\;\;\;\;\;\;a_{initial} & \;= & abs\;\left[FFT\left(I_{initial}\right)\right]\\ \;\;\;\;\;\;\;\;\;\;\;\;\;\;\;\varphi_{noise} & \;= & \left\{\;\begin{array}{cc} \tan^{-1}\;\left(\;\frac{S_{\varphi_{random}}}{C_{\varphi_{random}}}\;\right)+\;\pi & \mathrm{if}\;C_{\varphi_{random}}<0\;\\ \tan^{-1}\;\left(\;\frac{S_{\varphi_{random}}}{C_{\varphi_{random}}}\;\right)+\;2\pi & \mathrm{if}\;S_{\varphi_{random}}<\;0\;and\;C_{\varphi_{random}}>\;0\\ \tan^{-1}\;\left(\;\frac{S_{\varphi_{random}}}{C_{\varphi_{random}}}\;\right) & otherwise \end{array}\right. \end{array}$$

where:

$$\begin{array}{c} S_{\varphi_{random}}=\sin\left(\varphi_{random}\right)\\ C_{\varphi_{random}}=\cos\left(\varphi_{random}\right). \end{array}$$

This procedure was applied to each frame of the dynamic movies while φ_*random*_ was kept constant within but varied between trials.

In the static condition, each frame was combined with the same *I_noise_* as its corresponding frame in the dynamic condition. This result was possible because the generation of φ_*random*_ was seed controlled and was therefore the same across conditions for identical levels of phase coherence. This procedure ensured that the low-level properties of each frame of the movies and the information revealed by the noise were identical in both conditions.

### Methods

As shown in Figure 1, the movies presented to the observers were characterized by their level of signal, with 0% signal corresponding with a completely noisy pattern and 100% signal with natural images. We determined the amount of signal presented using two different procedures across two sessions: a uniform and an adaptive sampling. Under uniform sampling conditions, participants were shown images masked by a random amount of noise that was sampled independently for each trial, participant, expression, and condition from a uniform distribution ranging between 0% and 100%. Although all participants were not necessarily presented with precisely the same percentage of noise, this approach allowed to evenly sample the whole space when considering all observers. Our hypothesis for the impact of the signal level on response accuracy was an S-shaped response, where low and high percentages of signal would lead to a predominance of wrong and correct answers, respectively. However, the level of signal at which the transition between these two extremes would occur was unknown. Therefore, to capture this moment more precisely, we adopted an adaptive sampling approach that modelled the amount of signal to be shown based on observers’ previous responses. To this aim, on a separate testing session, we first presented 20 trials with a signal level drawn from a uniform distribution. Then we used curve fitting and inverse transform sampling to increase the likelihood of sampling a signal percentage around the point of the curve's maximum slope. This process was repeated after each of the remaining trials. Importantly, this adaptive sampling procedure was implemented for each participant and expression separately.

The stimuli were shown on a color liquid-crystal display with a resolution of 1,440 × 900 pixels and a refresh rate of 60 Hz. The experiment was programmed in MATLAB (2014) using the Psychophysics Toolbox (Brainard & Vision, 1997; Kleiner et al., 2007).

### Procedure

We informed our participants that they would be exposed to faces expressing different emotional expressions on a computer screen and that their task would be to categorize them as accurately as possible, according to the six basic facial expressions: anger, disgust, fear, happiness, sadness, and surprise. All participants sat 65 cm away from a computer screen in a quiet room at the University of Fribourg. Each trial started with a white fixation cross presented for 500 ms on a grey background at the centre of the screen. Facial expressions were then presented in random order at the centre of the screen, one at a time, for 1 second each and at a signal strength estimated by the uniform or the adaptive sampling (for a schematic representation of the procedure, see Figure 1). The same presentation time was used in both conditions. We decided to use a 1-second stimulus presentation time as it was previously used in several different studies with dynamic faces (Adolphs et al., 2003; Recio, Schacht, & Sommer, 2013; Richoz et al., 2015). After each stimulus, a response window appeared at the centre of the screen and remained there until the participant gave their answer by pressing the correct key on a labelled keyboard. We gave the observers as much time as needed to familiarize themselves with the different possible responses and their corresponding keys and told them that reaction time was not important for the current experiment. No feedback was given to the observers for their answers. If participants did not know the answer or did not have enough time to judge an expression, they could press an, “I don't know”–labelled key on the keyboard. Such a key was proposed to the participants to reduce the noise and response bias produced by the absence of this response possibility. Participants performed 768 trials presented with the uniform sampling of the noise and 768 trials with the adaptive sampling of the noise, for a total of 1,536 trials divided into 2 different sessions that took place on different days. Each session lasted for approximately 50 minutes. The 768 trials included 8 identities expressing 6 expressions 8 times in static and dynamic conditions (8 × 6 × 8 × 2). The 768 trials were divided into 10 blocks of 77 stimuli (75 for the last one). The stimuli were not blocked by condition, and each block presented a random ratio of dynamic and static faces. Before starting the testing phase, participants completed 12 practice trials in each condition.

### Data analysis

Data analysis was performed in R (The R Foundation for Statistical Computing, Vienna, Austria). First, we computed the average accuracy percentage by using a 2% signal window, from 0% to 2% of signal to 98% to 100% of the signal, which resulted in 50 bins. This procedure was performed twice: once for the dynamic and the static condition independently of the expressions and once for each condition and expression separately.

The binned data were then fitted using a three-parameter Weibull type II curve. Using each model, we estimated the psychometric curve as a function of signal at a resolution of 0.01% (i.e., resulting in 10,001 points between 0% and 100% of signal). These values were used to compare dynamic and static conditions (i.e., dynamic–static) in terms of accuracy, as well as to determine the amount of signal at which the greatest difference between both conditions occurred. This dynamic–static difference was subsequently compared across expressions.

Additionally, we estimated and compared between conditions the amount of signal required to reach 99% of the upper asymptote (i.e., ceiling point) and to surpass chance level (i.e., 1/6). Finally, using the estimated models’ parameters, we differentiated each curve and extracted the slope as a function of signal at a resolution of 0.01. Using these values, we determined the maximum slope, which we then compared between conditions for each expression separately.

To determine the statistical significance of the comparisons, we used percentile bootstrap. First, we sampled subjects with replacement and binned the data. We then fitted the Weibull type II curve, estimated the same measures as for the original data, and computed the differences of interest (i.e., dynamic–static or between-expression differences). This process was repeated 9,999 times allowing us to build a 95% confidence interval (CI) for each difference, which was considered as statistically significant if its CI did not encompass 0. Importantly, the CI was adjusted using Bonferroni correction to account for multiple comparisons when testing each of the six basic expressions and the overall condition (i.e., 7 contrasts) or cross-comparing facial expressions (i.e., 15 contrasts). This correction was implemented by dividing the alpha level of 5% in the CI by the number of contrasts performed. In this case, the CI will correspond to 100, the corrected alpha level. To simplify the reading, we refer to this statistical adjustment for the number of inferences made as the corrected CI.

Finally, to better understand the data, we used observers’ binned data to determine, in percentages, how often each expression was recognized as the correct one or as another one. These values were used to build confusion plots (see Supplementary Figures S1 and S2) and matrices to display participants’ choices as a function of the condition, expression, and amount of signal presented. All data used in statistical analyses and represented in figures are available online on the Open Science Framework repository at: https://osf.io/3269r/?view_only=23d291246d3644f898a21c08729d6dae.

## Results

**Figure 2.**
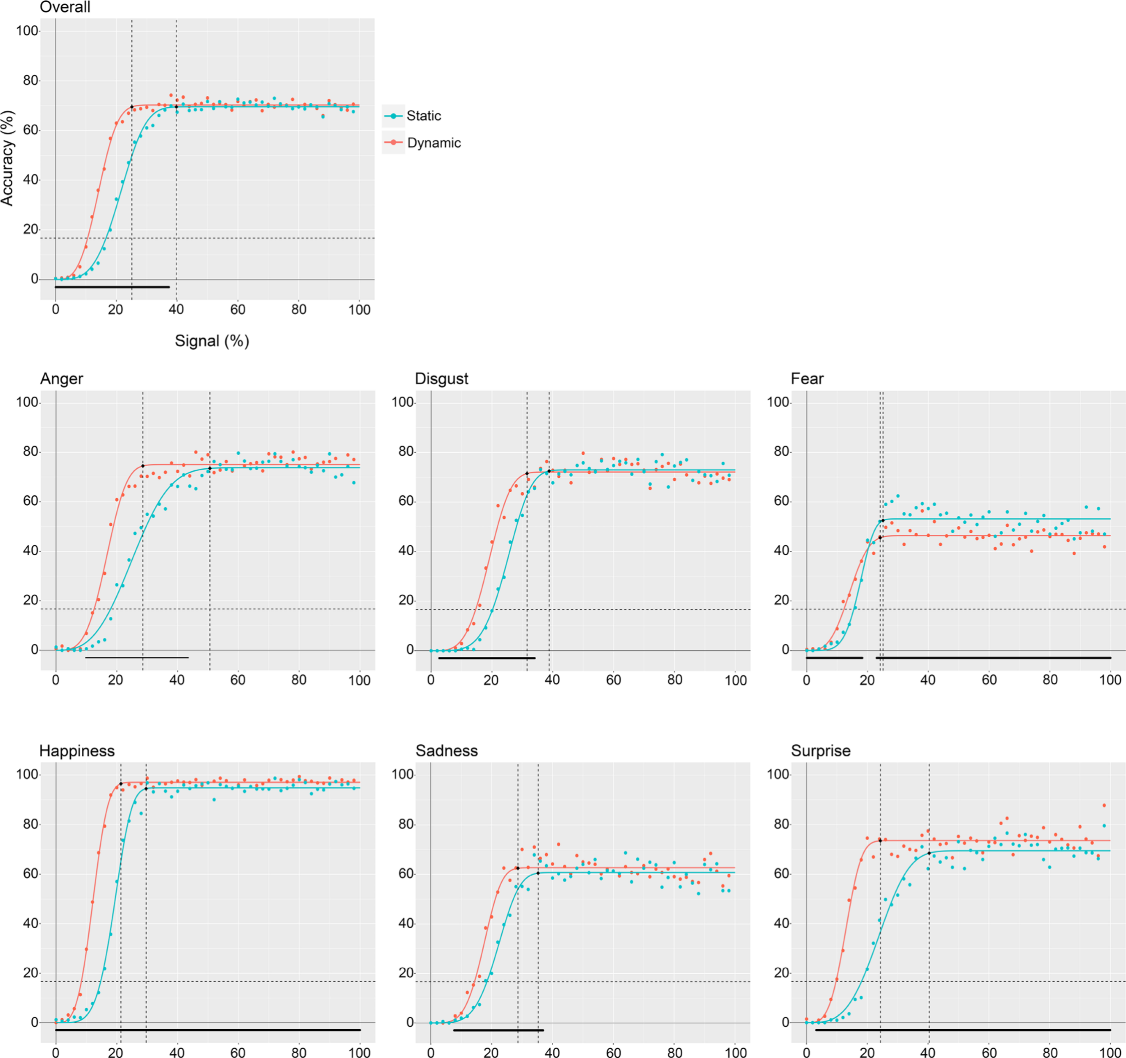
Raw data and curve fitting for dynamic and static conditions overall and for each expression separately. Dots represent raw data, while lines represent fitted curves. The vertical dotted lines mark the amount of signal needed to reach the ceiling point (i.e., 99% of the maximum performance). The horizontal dotted line indicates chance level. Triangles below the *x* axis mark the presence of a significant difference in terms of accuracy between dynamic and static conditions.

Figure 2 shows curve fitting across expressions and conditions. Data follow the expected S-shaped patterns, as accuracy grows from 0% when 0% of signal is shown, to a ceiling performance when 100% of signal is presented. Visual exploration of the raw data and curve fitting shows that the ceiling points as well as the rate of growth and quantity of signal required to accomplish this transition vary across both expressions and conditions (Figure 2).

As mentioned elsewhere in this article, to evaluate differences between conditions and expressions statistically, we used percentile bootstrap to build a corrected CI. We considered a difference significant if its corrected CI did not encompass zero. This statistical processing was applied on the data predicted by the curve that was fitted on the raw data.

### Dynamic vs. static FER performance across signal percentage: The dynamic advantage

The difference in FER performance between dynamic and static conditions was computed by subtracting, for each binned signal percentage, accuracy in the static condition from modelled accuracy in the dynamic condition. Consequently, any positive difference reflects a recognition advantage of dynamic over static expressions, and any negative difference an advantage of static over dynamic expressions.

**Figure 3.**
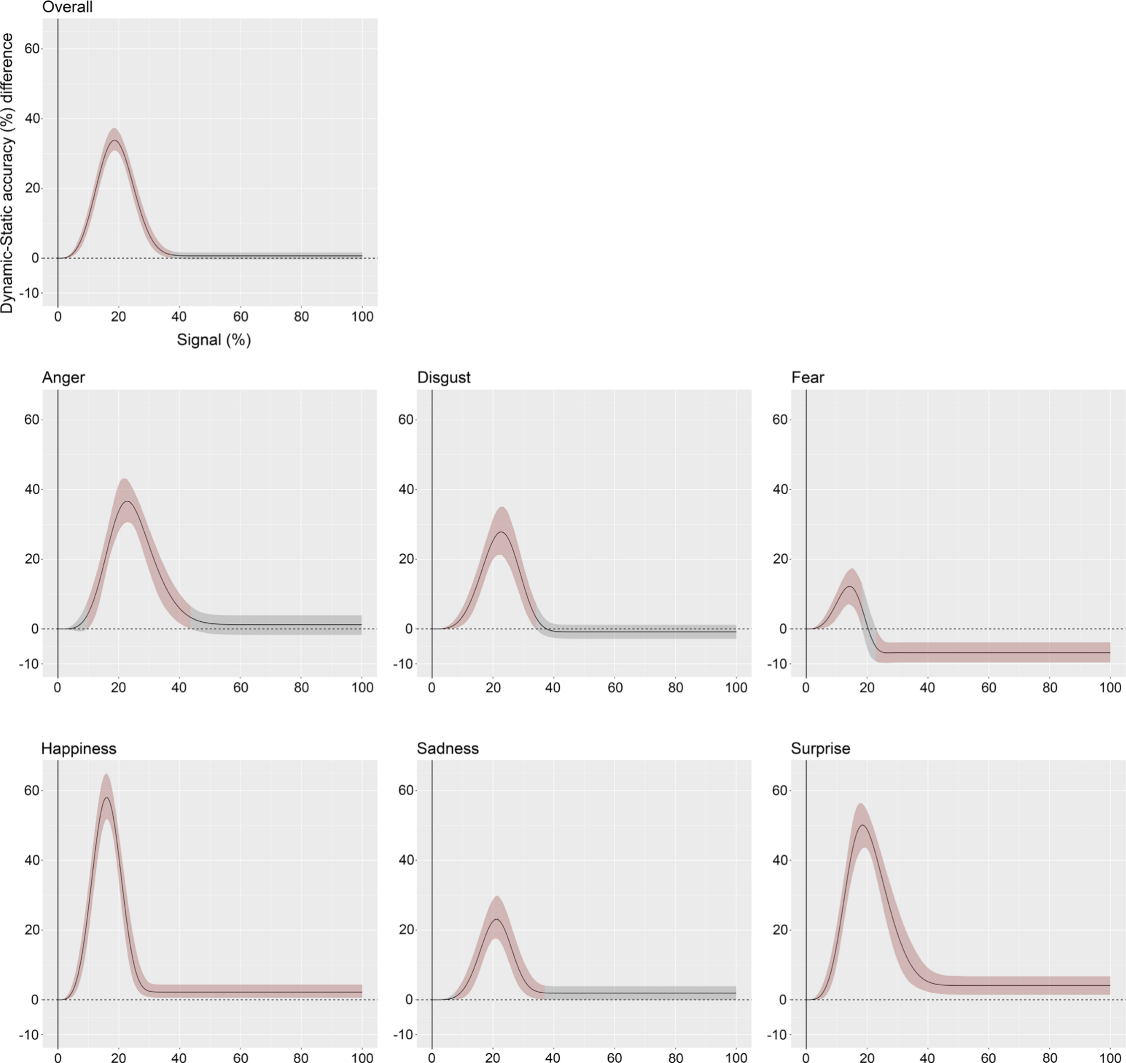
Dynamic–static curve difference overall and across each expression separately as a function of signal percentage. Lines show the dynamic-static difference between fitted curves as a function of signal percentage. Shaded areas around the curve indicate the corrected CI. Red shading indicates that the CI does not include 0 (i.e., statistically significant), which is illustrated here as the horizontal dashed line. Gray shading indicates that the CI includes 0, and the difference is therefore nonsignificant. CI = confidence interval.

**Figure 4.**
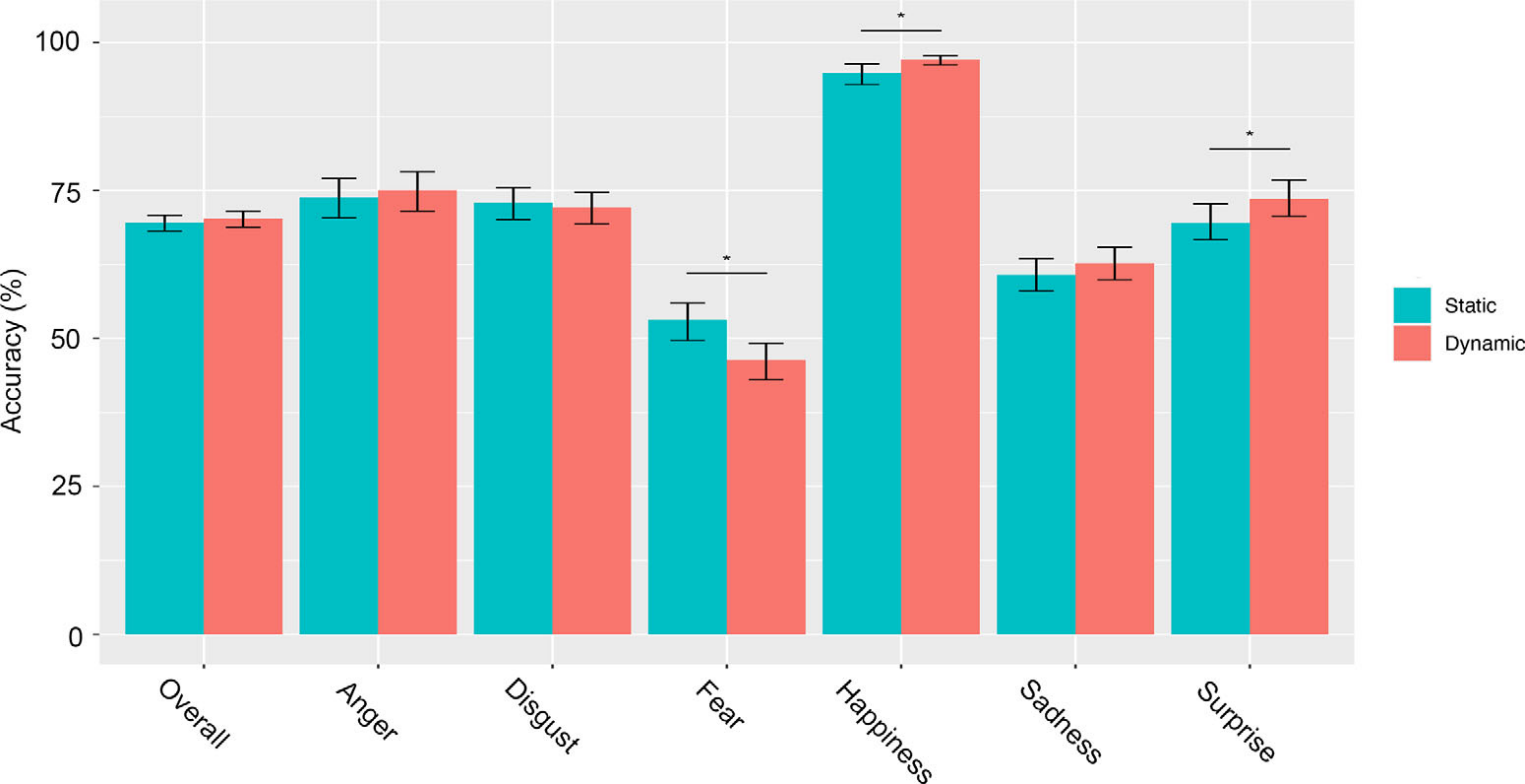
Accuracy level at 100% of signal. Accuracy levels in the dynamic and static conditions are reported overall and for each expression independently. Error bars represent the 95% CI. The * indicates a significant difference between conditions based on the corrected CI. CI = confidence interval.

Overall, comparisons between fitted curves show that a significant dynamic advantage emerges as early as 0.01% of signal. The advantage then grows and peaks at 18.6% of signal before decreasing and disappearing from 37.36% of signal onward (Figure 3). Although a similar pattern in the dynamic advantage is found across all expressions, some differences can be observed. The advantage onset occurs later in terms of signal for anger (9.65%), disgust (2.65%), sadness (7.64%), and surprise (3.16%) (Figure 3). As reported in Table 1, the amount of signal at which the greatest dynamic advantage occurs also varies across expressions. Additionally, for two expressions out of six (i.e., happiness and surprise) the dynamic advantage persists until 100% of signal, and for one expression (i.e., fear), it converts into a static advantage from 22.95% of signal onward (Figure 3, Figure 4, and Table 2).

**Table 1.** Magnitude of the dynamic advantage and level of signal at which it occurs.

	Magnitude of the maximum dynamic advantage	Signal at maximum dynamic advantage
Overall	33.83 [30.91–37.34]	18.60 [18.03–19.26]
Anger	36.66 [30.68–42.91]	22.76 [21.50–23.79]
Disgust	27.85 [21.23–35.13]	22.73 [21.62–23.66]
Fear	12.24 [7.01–17.02]	14.23 [13.18–15.91]
Happiness	58.02 [51.79–64.88]	15.98 [15.44–16.59]
Sadness	23.05 [17.58–29.82]	21.13 [20.03–22.17]
Surprise	50.10 [43.43–56.16]	18.50 [17.60–19.46]

*Note.* For the amplitude of the maximum dynamic advantage, we report the corrected CI. For the signal at which such advantage occurs, we report the 95% CI. CI = confidence interval.

**Table 2.** Measures [95% CI] estimated from the fitted curves.

	Accuracy at 100% signal	Maximum slope	Signal at ceiling point (99% of maximum performance)	Signal at chance level
Static				
Overall	69.57 [68.17, 70.82]	4.17 [3.86, 4.50]	39.77 [34.13, 41.71]	16.60 [16.04, 17.26]
Anger	73.79 [70.38, 77.04]	3.02 [2.70, 3.51]	50.63 [43.27, 58.35]	17.96 [17.10, 19.04]
Disgust	72.95 [70.10, 75.51]	4.62 [4.16, 5.19]	38.80 [36.17, 43.46]	20.21 [19.18, 21.28]
Fear	53.13 [49.65, 55.98]	5.42 [4.33, 7.20]	25.09 [22.79, 28.99]	15.45 [14.36, 16.63]
Happiness	94.84 [92.91, 96.41]	8.29 [7.15, 9.71]	29.64 [27.22, 32.84]	14.69 [13.96, 15.48]
Sadness	60.76 [58.01, 63.52]	4.20 [3.57, 4.94]	35.27 [31.54, 29.74]	18.45 [17.45, 19.62]
Surprise	69.49 [66.69, 72.75]	3.47 [2.95, 4.15]	40.38 [37.48, 50.8]	18.11 [17.18, 19.11]
Dynamic				
Overall	70.26 [68.82, 71.53]	5.61 [5.15, 6.27]	25.11 [23.65, 30.16]	10.55 [10.02, 11.2]
Anger	75.04 [71.52, 78.21]	5.67 [4.72, 7.27]	28.58 [25.02, 34.32]	12.56 [11.65, 13.57]
Disgust	72.11 [69.33, 74.67]	5 [4.15, 6.15]	31.54 [28.53, 37.6]	14.66 [13.8, 15.66]
Fear	46.38 [43.02, 49.2]	3.67 [3.04, 4.96]	24.17 [21.14, 30.83]	12.23 [11.34, 13.65]
Happiness	97.07 [96.23, 97.81]	9.81 [8.63, 11.30]	21.35 [19.63, 24.93]	8.35 [7.69, 9.10]
Sadness	62.70 [59.93, 65.43]	5.31 [4.51, 6.51]	28.65 [25.05, 31.67]	14.38 [13.38, 15.45]
Surprise	73.59 [70.62, 76.79]	7.09 [5.88, 8.75]	24.32 [19.85, 26.79]	9.72 [8.98, 10.49]
Dynamic–static differences				
Overall	0.69 [−0.30, 1.62]	**1.44 [0.83, 2.29]**	**−14.66 [−18.37, −4]**	**−6.05 [−6.70, −5.43]**
Anger	1.24 [−1.70, 3.90]	**2.65 [1.32, 5.09]**	**−22.05 [−34.61, −9.55]**	**−5.40 [−6.91, −3.99]**
Disgust	−0.84 [−2.82, 1.2]	0.38 [−1.07, 2.21]	−7.26 [−15.17, 1.98]	**−5.55 [−7.07, −4.02]**
Fear	**−6.75 [−9.59, −3.81]**	−1.75 [−4.09, 0.15]	−0.92 [−7.05, 8.03]	**−3.22 [−4.19, −1.89]**
Happiness	**2.23 [0.6, 4.39]**	1.52 [−0.94, 4.18]	**−8.29 [−13.54, −1.92]**	**−6.34 [−7.49, −5.18]**
Sadness	1.94 [−0.09, 3.87]	1.11 [−0.16, 2.80]	**−6.62 [−14.7, −0.45]**	**−4.07 [−5.56, −2.89]**
Surprise	**4.09 [1.48, 6.72]**	**3.62 [1.79, 6.01]**	**−16.06 [−33.02, −11.31]**	**−8.39 [−9.96, −6.91]**

*Note*. For static and dynamic FER performance, we report the 95% CI. For dynamic–static differences, we report CI after alpha Bonferroni correction for multiple comparisons (i.e., the corrected CI). Bold indicates significant dynamic–static differences. A positive difference in accuracy and slope and a negative difference in signal both indicate an advantage for the dynamic condition. CI = confidence interval.

**Figure 5.**
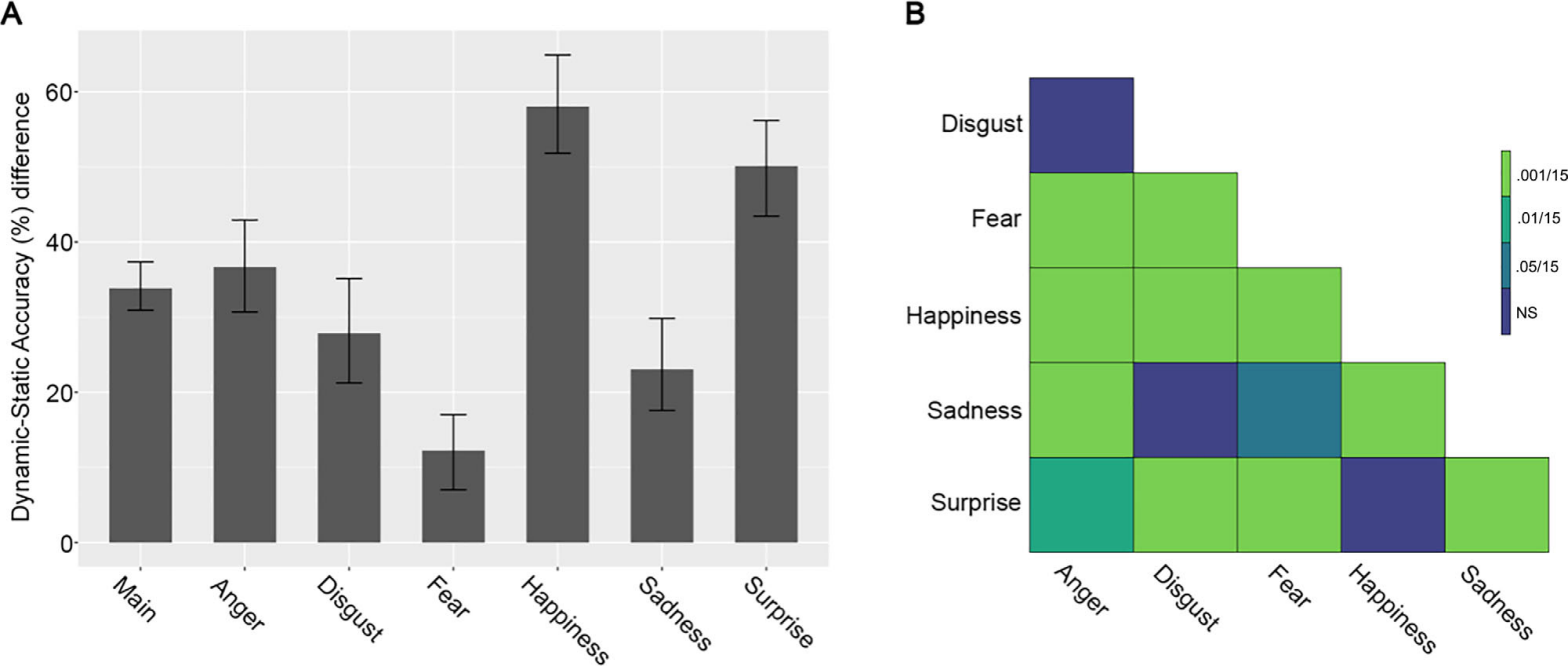
Dynamic advantage across expressions and cross-expression comparisons. (**A**) Bar plots represent the magnitude of the maximum dynamic advantage for each expression. Error bars represent the corrected CI. (**B**) The matrix represents the cross-expression comparisons of the magnitude of the maximum dynamic advantage. The significance of the contrasts was determined using the corrected CI and are color coded according to different alpha thresholds. CI = confidence interval.

Across all expressions, the largest dynamic advantage was observed for happiness (mean = 58.02%; 95% CI [51.79, 64.88]) and the smallest for fear (mean = 12.24%; 95% CI [7.01, 17.02]) (Figure 3, Figure 5A, and Table 1). Comparisons between expressions revealed that the magnitude of the dynamic advantage was significantly different across all of them, except for the disgust–anger, disgust–sadness, and happiness–surprise contrasts (Figure 5B). Assessing the percentage of signal at which the maximum dynamic advantage occurs shows that it first emerges for fear, followed by happiness, and surprise and it appears last for sadness, anger, and disgust (Table 1).

### Signal needed to reach the ceiling point (i.e., 99% of the maximum recognition performance)

As illustrated in Figure 2, participants’ accuracy shows an initial increase as a function of signal percentage, followed later by a plateau. To assess the minimum amount of signal required by participants to reach their maximum recognition performance (i.e., the ceiling point), we determined, for each expression and condition, the signal percentage at which performance reached 99% of the curve's upper asymptote.

**Figure 6.**
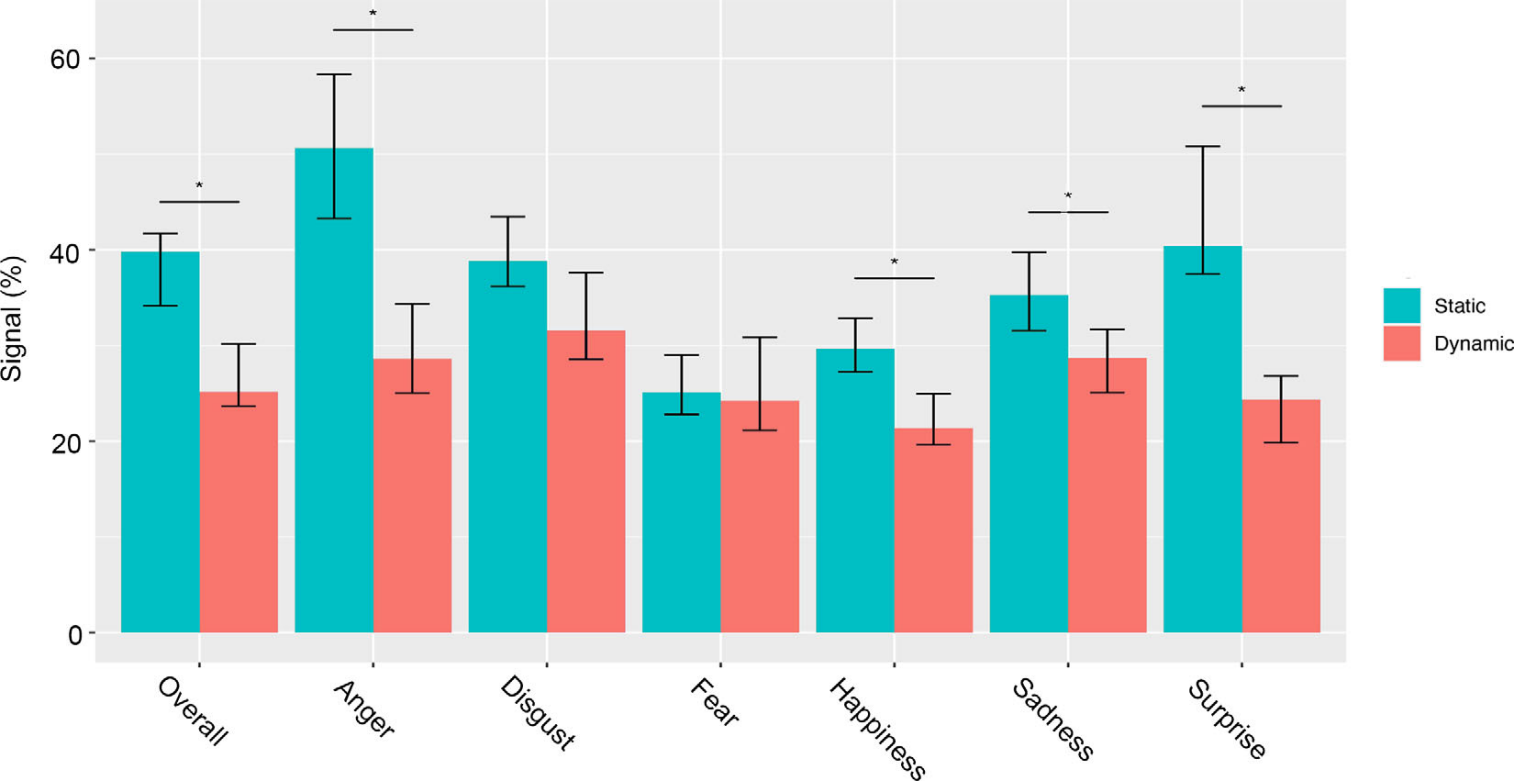
Minimum amount of signal needed by observers to reach ceiling points. The minimum amount of signal needed to reach the ceiling points (i.e., 99% of the maximum recognition performance) in both dynamic and static conditions are reported overall and for each expression independently. Error bars represent the 95% CI. The start indicates significant differences between conditions based on the corrected CI. CI = confidence interval.

**Figure 7.**
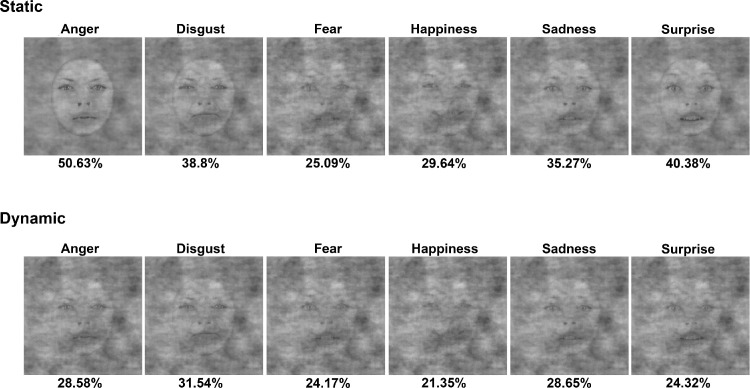
Minimum amount of signal needed by observers to reach the ceiling point (i.e., 99% of their maximum performance). Each expression is displayed with the level of signal at its ceiling point (i.e., 99% of maximum performance). Top and bottom rows illustrate the static and dynamic conditions, respectively.

Results show that both overall and for each expression independently, except for fear and disgust, participants needed significantly less signal to reach 99% of their maximum recognition performance in the dynamic condition (Figures 6, 7). More specifically, this difference was significant overall (*M* = −14.66, corrected CI [−18.37, –4.00]) and for anger (M = –22.05, corrected CI [−34.61, –9.55]), happiness (M = –8.29, corrected CI [−13.54, –1.92]), sadness (M = –6.62, corrected CI [−14.7, –0.45]), and surprise (M = –16.06, corrected CI [−33.02, –11.31]) (Figure 6 and Table 2).

### Signal needed to surpass chance level

**Figure 8.**
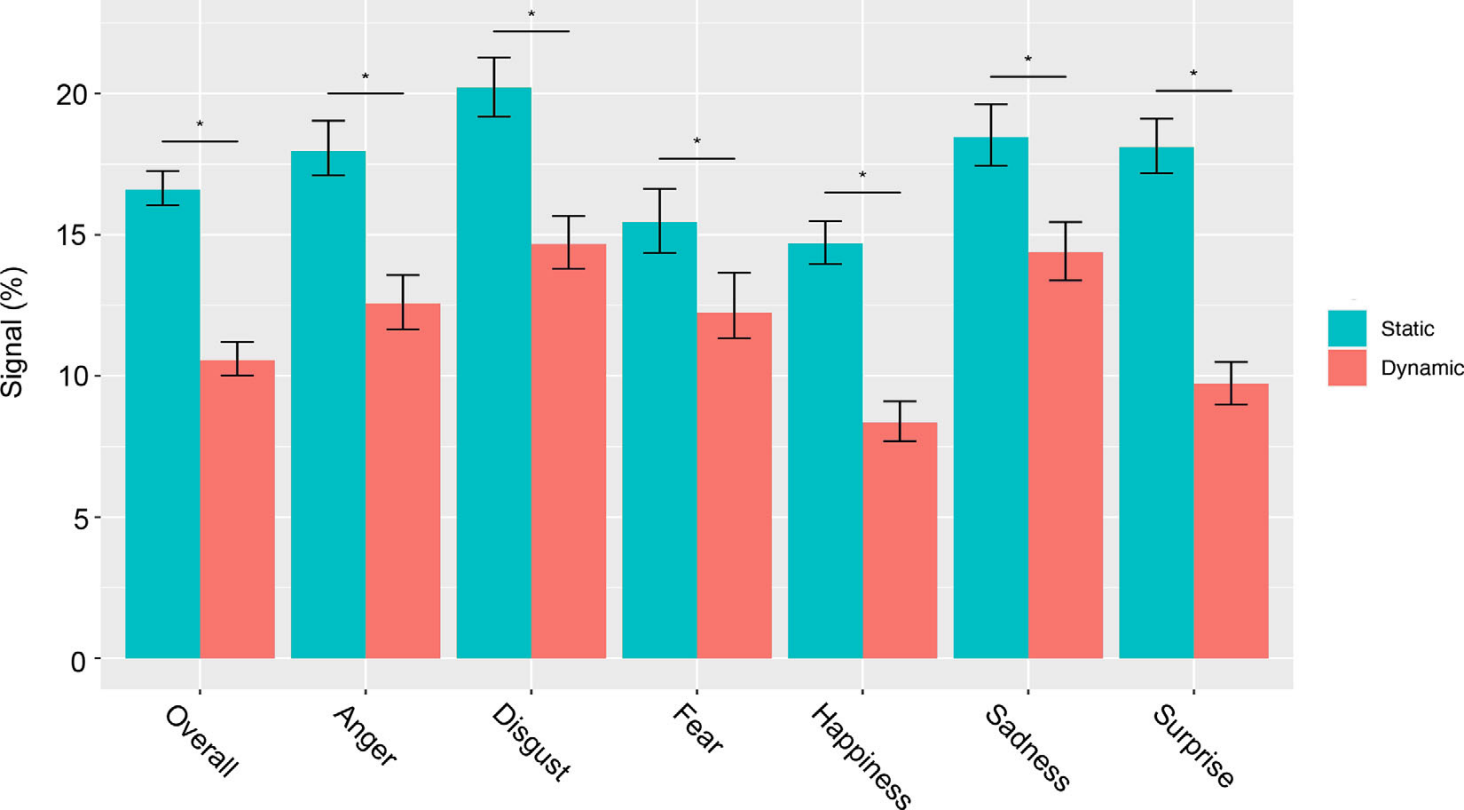
Minimum amount of signal needed by observers to surpass chance level. The minimum amount of signal needed**,** surpass chance level in both dynamic and static conditions is reported overall and for each expression independently. Error bars represent the 95% CI. The * indicates significant differences between conditions based on the corrected CI. CI = confidence interval.

Finally, we examined the amount of signal required to surpass chance level in the dynamic and static conditions. We defined chance level performance as the scores at 16.66%, which corresponds with one over six possible answers. The first score above 16.66% was considered as the value surpassing chance level. Results show that both overall and for each expression individually, observers needed significantly less signal to reach chance level in the dynamic compared with the static condition (*p* < 0.001) (Figure 8 and Table 2).

### Growth rate across dynamic and static conditions

**Figure 9.**
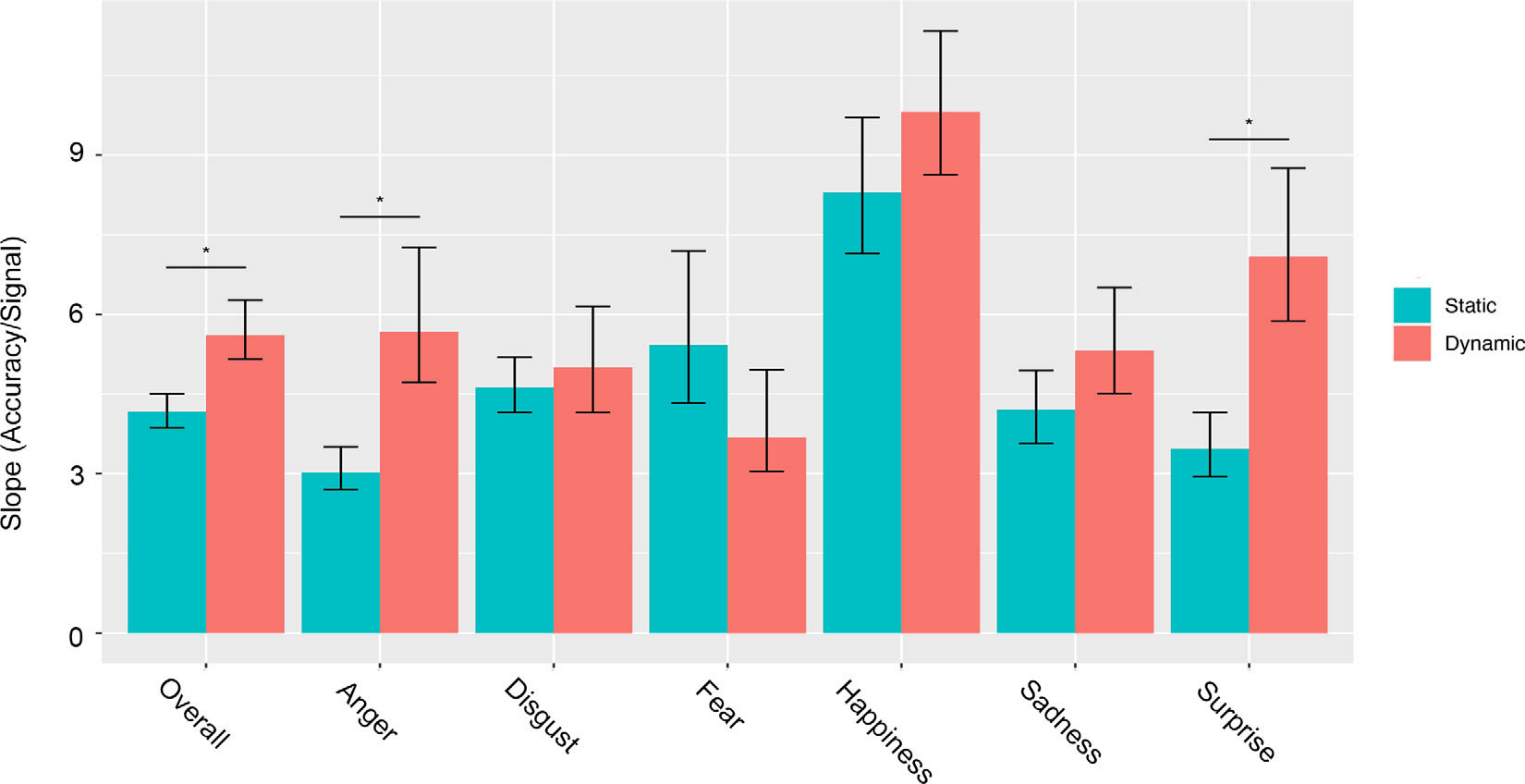
Maximum slope across conditions for each expression and overall. The maximum slopes of the fitted curves in the dynamic and static conditions are reported overall and for each expression independently. Error bars represent the 95% CI. The * indicates significant differences between conditions based on the corrected CI. CI = confidence interval.

To assess the rate of maximum accuracy increase as a function of the signal presented, we extracted the slope of the curve for each expression in each condition separately. Results show that overall and for each expression separately, except for fear, signal increase led to a steeper accuracy increase in the dynamic compared with the static condition (Figure 9). However, the statistical comparison of the maximum slope between conditions was significant only when all expressions were pooled together (M = 1.44, corrected CI [0.83, 2.29]), and only for anger (M = 2.65, corrected CI [1.32, 5.09]) and surprise (M = 3.62, corrected CI [1.79, 6.01]) when the expressions were considered separately (Table 2).

## Discussion

This study provides a novel fine-grained parametrical mapping of young adults’ ability to categorize static and dynamic expressions from low to full signal strength. By using an innovative psychophysical approach, we parametrically and randomly manipulated the quantity of signal available to the observers. We relied on a database of stimuli that was created by Gold et al. (2013), who ensured that the low-level physical information carried by static and dynamic faces was equal in both conditions. Moreover, because all stimuli were equated for their low-level properties, we could identify the genuine quantity of signal required for our observers to effectively categorize the six basic expressions in static and dynamic conditions. This precise and novel approach allowed us to clarify whether the very limited to noninexistent advantage for the recognition of dynamic expressions previously reported in young adults (e.g., Gold et al., 2013; Fiorentini & Viviani, 2011; Jiang et al., 2014; Richoz et al., 2018b) is rooted in a ceiling effect owing to the experimental conditions typically found in FER tasks.

### A dynamic advantage for all expressions with low signal

First and foremost, our findings revealed no overall beneficial effect of motion in healthy young adults when the stimuli were presented with 100% of phase signal, as recognition scores were very similar in both conditions. With these results, we replicate previous findings using identical (Gold et al., 2013) or similar stimuli (intense expressions) and methodological paradigms (optimal viewing conditions) (e.g., Bould & Morris, 2008; Jiang et al., 2014; Yitzhak et al., 2018). However, when examining each expression independently, our findings revealed a dynamic advantage with full signal strength for happiness and surprise. Although some prior studies (e.g., Gold et al., 2013) only examined the overall dynamic advantage without considering each expression individually, the current results are consistent with the ones we reported in a previous study revealing a dynamic over static advantage for happiness and surprise in healthy young adults (Richoz et al., 2018b). Thus, for young adults, optimal dynamic signals offer processing benefits only for a few expressions. For most emotions, the additional temporal information provided by dynamic faces such as muscular changes, temporal evolution, and velocity are not necessary for young adults to recognize them effectively when they are presented in optimal viewing conditions.

Second, we examined whether the near-optimal facial expression decoding system of healthy young adults is genuinely insensitive to the richness of dynamic signals or whether the very limited dynamic advantage observed in previous studies is rooted in a ceiling effect owing to the experimental settings typically found in FER tasks. In other words, we tested the idea that a dynamic advantage is present for all expressions but can only be revealed with the use of more sensitive suboptimal visual signals that can occur in everyday life (distance, occlusion, etc.). To do so, we parametrically and randomly manipulated the signal (0%–100%) of the facial expressions presented to the observers by using a computerized psychophysical technique to generate unique noise patterns for each frame of the dynamic movies. We then applied the identical noise patterns to the corresponding static frame to ensure that the information available at each level of signal was identical in both conditions. We found that all dynamic facial expressions were better decoded than their static counterparts when presented with low signal. This dynamic advantage appeared with as little as 1% of signal for some expressions, gradually increased, peaked, and decreased with increasing signals, disappearing entirely at 38% of signal. Only happiness and surprise showed a different pattern, with a dynamic advantage persisting until 100% of signal. Note also that the initial dynamic advantage observed for fear changed into a static advantage at approximately 22% of signal. These findings suggest that dynamic cues provide additional emotion-related information that facilitate the recognition of all six basic emotional expressions in suboptimal visual conditions. Importantly, in healthy young adults, motion-related cues are beneficial for the recognition of anger, disgust, fear, and sadness only when static information is insufficient, compensating for the deleterious consequences of degraded or missing visual information.

Two perceptual processes might explain how facial dynamics improve emotion recognition in suboptimal situations. First, the saliency of change may naturally drive the attention of observers toward the diagnostic information in a bottom-up fashion (i.e., the mouth for happiness), while with static expressions, participants are required to direct their attention toward those facial features based on top-down internal representations. Secondly, motion signals, such as the direction of change, the temporal evolution of an emotional expression, the velocity of muscular changes and contractions, provide additional diagnostic information that might be critical to support adequate expression categorization in nonoptimal visual conditions (Kamachi et al., 2001; Kamachi et al., 2013; Yitzhak et al., 2018).

In addition, several neuroimaging studies that have examined the neural underpinnings of the dynamic advantage have shown that dynamic expressions involve dissociable neural pathways and elicit broader activations compare to static expressions (e.g., Johnston, Mayes, Hughes, & Young, 2013; Kessler et al., 2011; LaBar, Crupain, Voyvodic, & McCarthy, 2003; Paulmann, Jessen, & Kotz, 2009; Sato et al., 2004; Schultz & Pilz, 2009; Trautmann, Fehr, & Herrmann, 2009). For example, Sato et al. (2004) have reported enhanced activations to dynamic compared with static displays in right-lateralized occipital and temporal cortices comprising the inferior occipital gyri, middle temporal gyri, and fusiform gyri. In contrast, the perception of static displays has been shown to activate a network of motor, prefrontal, and parietal regions, typically involved in motor imagery (Kilts et al., 2003). More recently, Liang et al. (2017) have shown that dynamic compared with static expressions were associated with higher recognition accuracies and more robust neural responses in face-selective areas (occipital face area, fusiform face area, posterior superior temporal sulcus), as well as in motion-sensitive regions. These findings suggest that domain–general motion-sensitive areas that are not face specific are also strongly involved in decoding dynamic facial expressions. Observers’ enhanced ability to accurately decode dynamic expressions presented with a very low signal could be explained by the larger and more sensitive cortical network dedicated to their processing as compared with static expressions.

Note that our behavioral findings are in line with a small number of studies that have investigated static and dynamic FER in suboptimal visual situations with degraded or blurred faces (Ehrlich et al., 2000; Kätsyri & Sams, 2008; Wallraven et al., 2008). For instance, using computer-animated faces, Wallraven et al. (2008) revealed that motion cues enhanced the recognition of facial affects when texture or shape information was systematically degraded or blurred. If dynamic cues were not provided, degrading face information significantly affected expression recognition. However, one major issue of altering spatial frequency information (i.e., blurring faces) is that the recognition of some facial expressions can be more affected than others, because the diagnostic spatial frequencies are different across emotions (Plouffe-Demers et al., 2019; Schyns, Petro, & Smith, 2009; Tian et al., 2018). To overcome this limitation, in the current study, we used a psychophysical approach that normalized spatial frequency information for all expressions and experimental conditions. This methodological choice provides a more reliable view of how dynamic cues offer processing benefits for the recognition of all six basic facial expressions on the continuum from low to full-strength signal and may help to clarify past inconsistencies observed across studies.

### Maximum sensitivity to dynamic signals

Having established a dynamic advantage for the recognition of all facial expressions with low signal, we then estimated for each expression the maximum sensitivity to dynamic signals by determining the point at which the dynamic gain reaches its maximum before declining. We also estimated the quantity of signal necessary to reach this peak. To the best of our knowledge, this study is the first that has effectively quantified the strength of the dynamic gain for each expression and the availability of visual information at this point. We observed the strongest dynamic gain for happiness followed by surprise with differences in accuracy of 58% and 50% between static and dynamic conditions at the maximum dynamic gain, respectively. The quantity of signal necessary to reach the maximum dynamic gain was around 16% for happiness and around 19% for surprise. We observed the maximum dynamic gain for anger, disgust, and sadness between 21% and 23% of signal. Although significantly weaker than the dynamic gain for happiness and surprise, the differences in recognition performance between static and dynamic conditions at the maximum dynamic gain were 23% for sadness, 28% for disgust, and 37% for anger. Finally, we observed a very distinctive trajectory for fear with an early significant dynamic gain (12% differences in recognition accuracy) peaking at 14% of signal before shifting toward a significant static advantage between 23% and 100% of signal. Notably, the maximum dynamic gain observed for fear was significantly weaker than for all the other expressions.

The stronger dynamic advantage observed for some expressions over others might be explained by the diagnostic information embedded in the temporal evolution of these expressions. Our results here suggest that, among all expressions, the dynamics of happiness and surprise signals are the most informative. These signals might be particularly salient and act as attention grabbers when very low information is available to the observers, making them more detectable than static peak frames in suboptimal conditions. Kamachi et al. (2013) have also shown that quick dynamic events tend to be categorized as surprising or happy events, whereas slow or static events are more likely to be categorized as sad. The very strong dynamic advantage observed for happiness and surprise could thus also be due to the distinct temporal properties of these expressions being inherently dynamic and rapid (see also Bould et al., 2008; Jack et al., 2014; Yitzhak et al., 2018).

The maximum dynamic gain we found for anger and disgust was also strong—albeit significantly lower than for happiness and surprise. This finding was paired with a shift in the quantity of signal necessary to reach the maximum dynamic gain for these two expressions (i.e., more signal was needed). Previous studies have shown that the emotional expression of disgust is frequently confounded with anger (e.g., Recio et al., 2013; Richoz et al., 2018b; Rodger et al., 2015) that could be explained partly by the shared muscular action units between these two expressions (e.g., Poncet et al., 2021). More specifically, Jack et al. (2014) have shown that the confusion between anger and disgust occurs because both expressions share similar signals in early dynamics (nose wrinkler and lip funneler), suggesting that late dynamic signals are necessary to disambiguate those expressions. The signal shift we observed here could be accounted for by the necessity of revealing additional diagnostic information critical to disentangle the ambiguity triggered by those expressions. Additionally, we could also speculate that the early onset in the maximum dynamic gain observed for happiness–surprise over anger–disgust could rely on their frequency of exposure. During everyday life social interactions, we routinely smile to our friends, feeling joy or often expressing wonderment, a positive sentiment of surprise (Vrticka, Lordier, Bediou, & Sander, 2014). In contrast, we rarely face anger and disgust.

Interestingly, we observed a dynamic gain for sadness, an expression that has been previously shown to be better recognized through static face images (Bould et al., 2008; Recio et al., 2013; Richoz et al., 2018a; Widen & Russell, 2015) or when evolving very slowly (Kamachi et al., 2001; Recio et al., 2013; Richoz et al., 2018b; Widen & Russell, 2015). For instance, in a previous cross-sectional study, we failed to report a dynamic advantage for the categorization of sadness at any age (Richoz et al., 2018b). Ekman (2003) suggested that, among all expressions, sadness is the one lasting the longest over time, a property that may explain why slowness or stillness may increase recognition performance. Although this explanation might clarify the absence of a dynamic gain in optimal visual conditions and with intense expressions, the current results provide new evidence that the recognition of sadness benefits from dynamic cues when the diagnostic information is not fully available.

Finally, our findings revealed a very distinct trajectory for fear, supporting previous evidence that this expression has a special status within the framework of FER (Richoz et al., 2015; Rodger et al., 2015). We only observed a dynamic advantage for fear with a very low signal, peaking rapidly before converting into a static advantage. This initial dynamic advantage could be due to an increased saliency elicited by the wide and rapid opening of the eyes when a very low signal is available to the observers (see Liu et al., 2022). Our data also revealed a static advantage for recognizing fear between 23% and 100% of signal. Although counterintuitive at first sight, this static advantage could be explained by the diagnostic information conveyed by the emotional expression of fear over time. Using Bayesian classifiers, Jack et al. (2014) revealed that fear and surprise share similar muscular activations (upper lid raise, jaw drop) in early signaling dynamics, leading to systematic confusion between those two emotion categories. The critical diagnostic information (eyebrow raiser; Jack et al., 2014) that allows to accurately distinguish both expressions becomes fully available only in later signaling dynamics. Static expressions of fear, displaying the fully evolved late signaling dynamics for 1 second, are maximally informative and could thus be advantageous for the categorization of this expression (see also, Richoz et al., 2018b). Furthermore, given its unique evolutionary significance (i.e., indication of danger), the decoding of fear might recruit additional brain regions or faster neural pathways (e.g., the amygdala) that might shortcut the presumably longer processing trajectory of dynamic faces (e.g., Adolphs, 2008; Furl, Henson, Friston, & Calder, 2013). For instance, Furl et al. (2013) have shown that the amygdala plays a critical role in the decoding of static and dynamic fearful expressions by recruiting distinct brain areas in a context-sensitive fashion (form, or motion) to enhance and optimize their processing. With dynamic faces the amygdala targets the superior temporal sulcus and V5, both involved in the encoding of motion information (e.g., Pitcher et al., 2011; Schultz & Pilz, 2009), whereas with static expressions the amygdala selectively targets the fusiform face area, an area dedicated to the processing of facial identity (e.g., Haxby, Hoffman, & Gobbini, 2000) and static facial expressions (e.g., Ganel, Valyear, Goshen-Gottstein, & Goodale, 2005). These findings suggest that the amygdala guides and controls how socially salient information is visually encoded by modulating its connections to dorsal and ventral brain regions.

### Static and dynamic emotion recognition trajectories from low to full signal

To further examine the specific static and dynamic emotion recognition trajectories from low to full signal, we estimated for each expression the quantity of signal required to surpass chance level, defined as the first score above 16.66% (1/6 possible answers). Strikingly, we observed that the amount of signal necessary to surpass chance level was significantly higher for all expressions in the static compared with the dynamic condition. These findings further confirm the existence of a dynamic advantage for recognizing all facial expressions and reinforce the notion that dynamic faces are richer, ecologically more valid depictions of real-life face representations, enhancing recognition performance in suboptimal situations.

We also estimated a ceiling point that we defined as the value at which participants reached 99% of their maximum recognition performance and again determined the quantity of signal necessary to reach this point. Our findings revealed that more signal was required to reach the ceiling point in the static compared with the dynamic condition. When considering each expression individually, we could evidence that this was true for anger, happiness, and surprise. In addition, our data also evidenced that fear and happiness were the first two expressions to reach their ceiling points, regardless of the condition. In other words, less signal was required by the participants to reach their maximum recognition performance for these two expressions. From an evolutionary perspective, the emotional expression of fear transmits critical signals to detect dangers and avoid harmful situations. Therefore, the biological relevance and importance for human survival of this expression could explain why observers reached their maximum recognition performance very rapidly and needed less signal compared with the other expressions. As for happiness, the very early ceiling points observed in both conditions might be accounted for by the high frequency of exposure to this emotion during everyday life social interactions (Calvo, Avero, Fernández-Martín, & Recio, 2016), as well as to the orthogonal muscular activations elicited by this expression. This physiological pattern results in a very effective transmission of diagnostic facial information, hence leading to an early peak in maximum recognition accuracy. The dynamic advantage we observed for the decoding of facial expressions of emotion might arise from the integration of form and movement, as previously shown for face identification (Dobs, Bulthoff, & Schultz, 2016; O'Toole, Roark, & Abdi, 2002). Future studies manipulating these facial information properties are necessary to clarify this question.

Finally, to provide an estimate of the nature of emotion recognition process (i.e., categorical vs. continuous), we examined the steepness of increase in recognition performance across emotions and conditions as a function of signal. To do so, we extracted the slope of the curve for each expression in each condition. This procedure allowed us to examine how quickly the transition happens from very low to very high recognition accuracy in each condition. Overall, our data revealed a steeper increase in the dynamic compared with the static condition, suggesting that, with an increase of signal, participants quickly transition to much higher recognition performance with moving compared with still faces. When considering each expression separately, we observed a significantly steeper increase in the dynamic compared with the static condition for anger and surprise. Similar results were observed for all the other expressions, except for fear, yet the differences between conditions were not significant. This pattern of results posits that human observers are more sensitive to signal changes with dynamic compared with static expressions. For the latter, the increase of visual information more slowly benefits the participants.

Altogether, this last set of findings provides some novel insights into the unfolding of static and dynamic FER as a function of the amount of visual signal available, thereby clarifying some of the contrasted and discrepant results previously reported in the literature. As observed, the dynamic advantage depends on the very nature of the diagnostic information available. Driven by experience and shaped by evolutionary and ontogenetic dynamics, the human visual system is optimally tuned to successfully categorize all dynamic expressions in suboptimal situations. Our findings also support previous neurofunctional explanations suggesting the existence of distinct cortical pathways for processing static and dynamic face information (Duchaine & Yovel, 2015).

### Methodological considerations and future directions

In the current study, we used a computerized psychophysical paradigm to parametrically and randomly manipulate the signal of the static and dynamic expressions presented to the observers. We used uniform and adaptive sampling to determine the level of signal presented in each trial. Under uniform sampling conditions, the amount of noise was randomly sampled for each participant from a uniform distribution ranging between 0% and 100%. Although this methodological choice allowed us to examine the dynamic over static advantage across signal, it did not allow us to precisely determine participants perceptual thresholds for an effective recognition of the six basic expressions in static and dynamic conditions, especially not at the single subject level. Further studies implementing threshold-seeking algorithms are necessary to address this question and examine quantitative individual differences in static and dynamic facial signal use.

In addition, uniform and adaptive sampling procedures allowed us to limit the number of trials while evenly sampling the whole space when all observers were considered together. Yet, with such procedures, we did not get the same amount of data points across all the levels of signal. Therefore, in our future study, we will address this limitation by defining precisely specific levels of signal that will be presented to all our participants.

Note also that we cannot rule out that the noise patterns affected the perceptual strategies used by our participants to recognize the facial expressions. However, as mentioned elsewhere in this article, we generated the noise using a seed-controlled procedure, so that corresponding static and dynamic stimuli were assigned with the same noise patterns for identical signal levels. As such, if holistic strategies were affected by the noise patterns added to the stimuli, then the expressions would have been affected identically in static and dynamic conditions, given that the same noise patterns were used in both conditions. Future work will examine the fixation distribution of our participants to shed further light on facial information use during this task.

Finally, it would be interesting to examine whether the emotion-specific dynamic vs. static advantages observed in the current study generalize to other databases of facial expressions of emotion, populations (Quesque et al., 2022), and cultures (e.g., Caldara, 2017; Jack, Blais, Scheepers, Schyns, & Caldara, 2009).

## Conclusions

Prior studies investigating the dynamic advantage for the recognition of facial expressions of emotion have yielded contrasted findings, with some suggesting that dynamic cues provide processing benefits, while others suggesting they do not (Fiorentini & Viviani, 2011; Gold et al., 2013) or only in specific populations (Alves, 2013). To further clarify this question, we examined static and dynamic FER across signal (0%–100%) by parametrically and randomly manipulating the quantity of visual information available to the observers. Our findings revealed that facial movements provide additional cues for the recognition of all facial expressions in suboptimal situations (i.e., with low signal), allowing observers to disentangle the ambiguity triggered by insufficient, lacking, or degraded information. In contrast, in optimal situations, the visual system of healthy young observers is powerful enough to efficiently categorize static emotional expressions, with dynamic faces enhancing recognition performance only for happiness and surprise with 100% of signal. By extracting the slope of the curves, our analyses allowed us to further estimate the steepness of increase in recognition performance as a function of signal for each expression in each condition. We also examined the quantity of signal necessary to surpass chance level and to reach a ceiling point in recognition performance. Overall, we observed the steepest accuracy increment in the dynamic condition; less signal was also required to surpass chance level and to reach a ceiling point in recognition performance in this condition. Altogether, our findings confirm the existence of a dynamic advantage for the recognition of facial expressions of emotion, but also evidence that this advantage depends on the very nature of the visual information available. In line with evolutionary and ontogenetic perspectives as well as neurofunctional explanations, dynamic signals are more effective and sensitive than static inputs to reliably categorize facial expressions of emotion for all human observers. Our study increases our understanding of the recognition of static and dynamic expressions and offers a new approach to precisely map FER deficits in specific populations.

## Supplementary Material

Supplement 1
