# Supplementary material for: Recognizing facial expressions of emotion amid noise: A dynamic advantage
Source: J Vis. 2024 Jan 10;24(1):7. doi: 10.1167/jov.24.1.7 (PMC10790674; doi:10.1167/jov.24.1.7)
Supplement: Supplement 1 [file jovi-24-1-7_s001.pdf]

Figure S1

Confusion Plots for both Conditions for Anger, Disgust and Fear

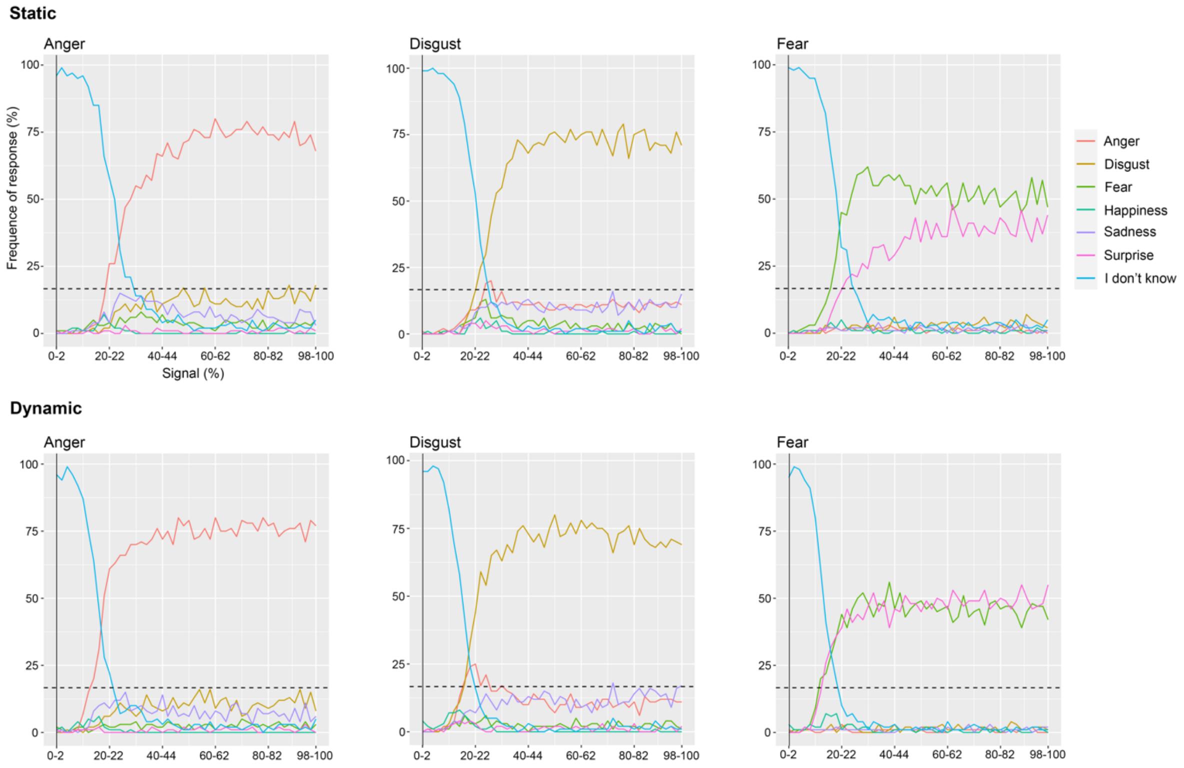

Note. The plots represent the frequency at which anger, disgust and fear were recognized by participants as such, as a different expression or as none (i.e., "I don't know"). Each color tags a specific facial expression. The horizontal dashed line represents the chance level (i.e., 1/6). The top and bottom rows represent the static and dynamic conditions, respectively

Figure S2

Confusion Plots for both Conditions for Happiness, Sadness and Surprise

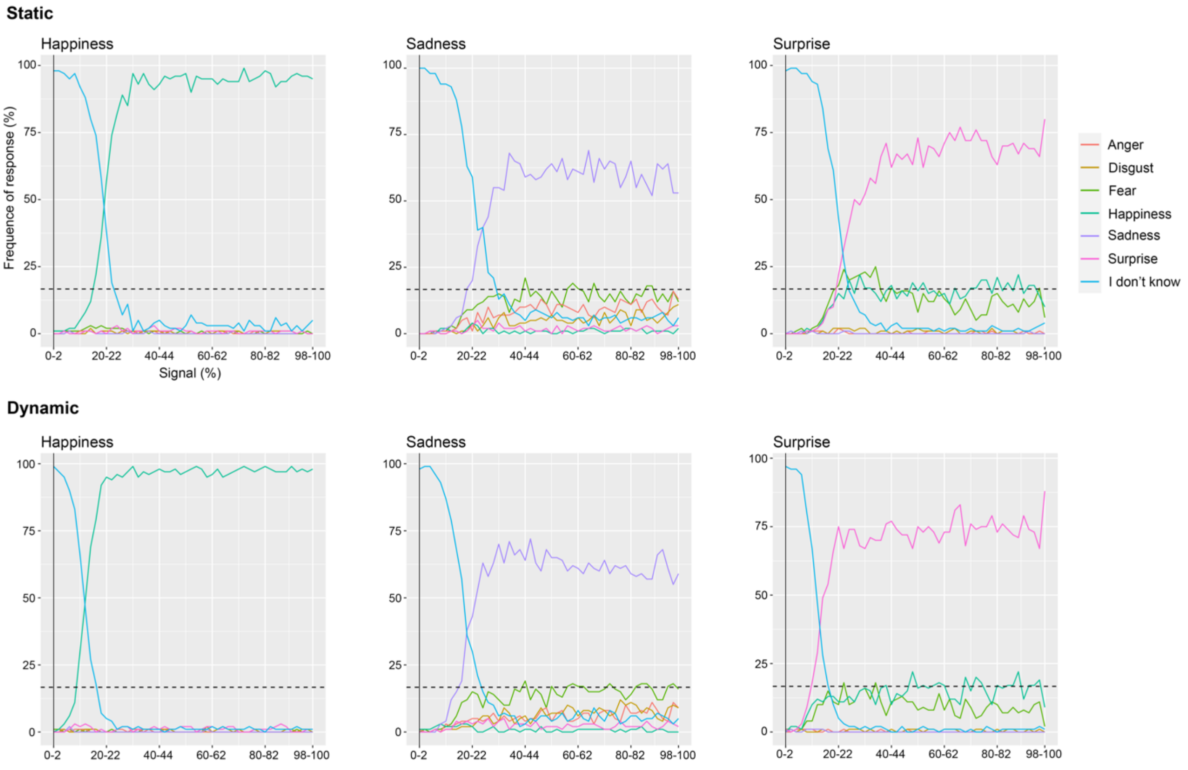

Note. The plots represent the frequency at which happiness, sadness and surprise were recognized by subjects as such, as a different expression, or as none (i.e., "I don't know"). Each color tags a specific facial expression. The horizontal dashed line represents the chance level (i.e., 1/6). The top and bottom row represent the static and dynamic conditions, respectively.
